# Shaping Viral Infection Outcomes via Organelle Remodeling

**DOI:** 10.1146/annurev-virology-092623-094221

**Published:** 2025-06-24

**Authors:** William A. Hofstadter, Ileana M. Cristea

**Affiliations:** Department of Molecular Biology, Princeton University, Princeton, New Jersey, USA

**Keywords:** virus, organelle remodeling, mitochondria, peroxisomes, plasma membrane, lipid droplets, endoplasmic reticulum, Golgi, secretory pathway, endocytosis, membrane contact sites, microscopy

## Abstract

Subcellular organelles are dynamic structures that tune their functions in conjunction with changes to their shapes and compositions. Each organelle has distinct structure-function relationships that change in response to diverse stimuli. Such remodeling events further affect organelle-organelle interaction networks facilitated by membrane contact sites, thereby activating rapid intra- and intercellular communication cascades. As viruses rely on repurposing the host cell machinery during infections, organelle remodeling is a fundamental facet and outcome of all viral infections. Some organelle remodeling events are unique to particular viruses, while others are shared by an array of viruses. Here, we review knowledge derived from this expanding yet still underexplored research area of infection-induced organelle remodeling. We focus on the molecular mechanisms used by viruses to temporally control organelle structure-function relationships. We highlight how organelle remodeling can inhibit host defenses or facilitate specific stages of a virus replication cycle, i.e., entry, replication, assembly, and spread.

## INTRODUCTION

Eukaryotic cells are stratified into distinct subcellular compartments, formed in part through membrane-bound organelles. This compartmentalization allows for many unique environments to exist within a cell, each of which can be specialized for different fundamental cellular processes. Organelles are not static structures and can toggle their functions in response to a range of intra- and extracellular stimuli. This modulation of organelle function occurs in conjunction with the dynamic remodeling of their composition, intra- and interorganellar interactions, localization, morphology, and/or abundance. As such, organelle remodeling is integrated into diverse cellular signaling pathways.

A prevalent means for altering organelle function is by tuning their cellular abundance. Many organelles can undergo coordinated proliferation through either fission, such as for endosomes (1), or de novo biogenesis, such as for peroxisomes (2). Conversely, organelle numbers can be decreased through fusion, such as for lipid droplets (3), or by degradation, such as for the Golgi apparatus (4). Organelles can also control their morphology and size to modulate their function. Modulation of organelle function by changing shape is frequently driven by the surface area-to-volume ratio (SA:V), a basic principle of biology. The larger a spherical object is, the lower the SA:V. For example, the endoplasmic reticulum (ER) spans nearly the entire cytoplasm. To increase its SA:V, this vast organelle forms thin, interconnected tubules that provide a large platform for membrane-associated functions, such as the exchange of molecules, without inflation of the matrix, which could unfavorably alter diffusion dynamics and decrease the efficiency of enzymatic reactions.

Organelles are fluid not only in their morphology and abundance but also in their macromolecular makeup, e.g., enrichment or depletion of lipids, proteins, and nucleic acids within organelle membranes or their matrix. Such changes in composition can affect organelle functionality by altering the biophysical properties of the organelle membrane or by directly dysregulating protein signaling pathways.

In addition to remodeling its own identity, an organelle can influence other organelles through expansive organelle-organelle interaction networks that coordinate cellular processes. Organelle-organelle interfaces, termed membrane contact sites (MCSs), are mediated by proteins that serve both to tether organelles together and to mediate the exchange of small molecules, such as lipids and ions (5, 6). MCSs facilitate many aspects of organelle remodeling, from promoting fission (7) and organelle maturation (8) to providing the lipids for membrane expansion (9). MCSs are prevalent structures, forming and disbanding in myriad cellular contexts. Having been traditionally investigated using microscopy and, more recently, by proteomics (10, 11), contacts have been found to form at 24 different organelle-organelle interfaces, and more than 7,000 MCS proteins have been identified to date (10).

**Figure 1:**
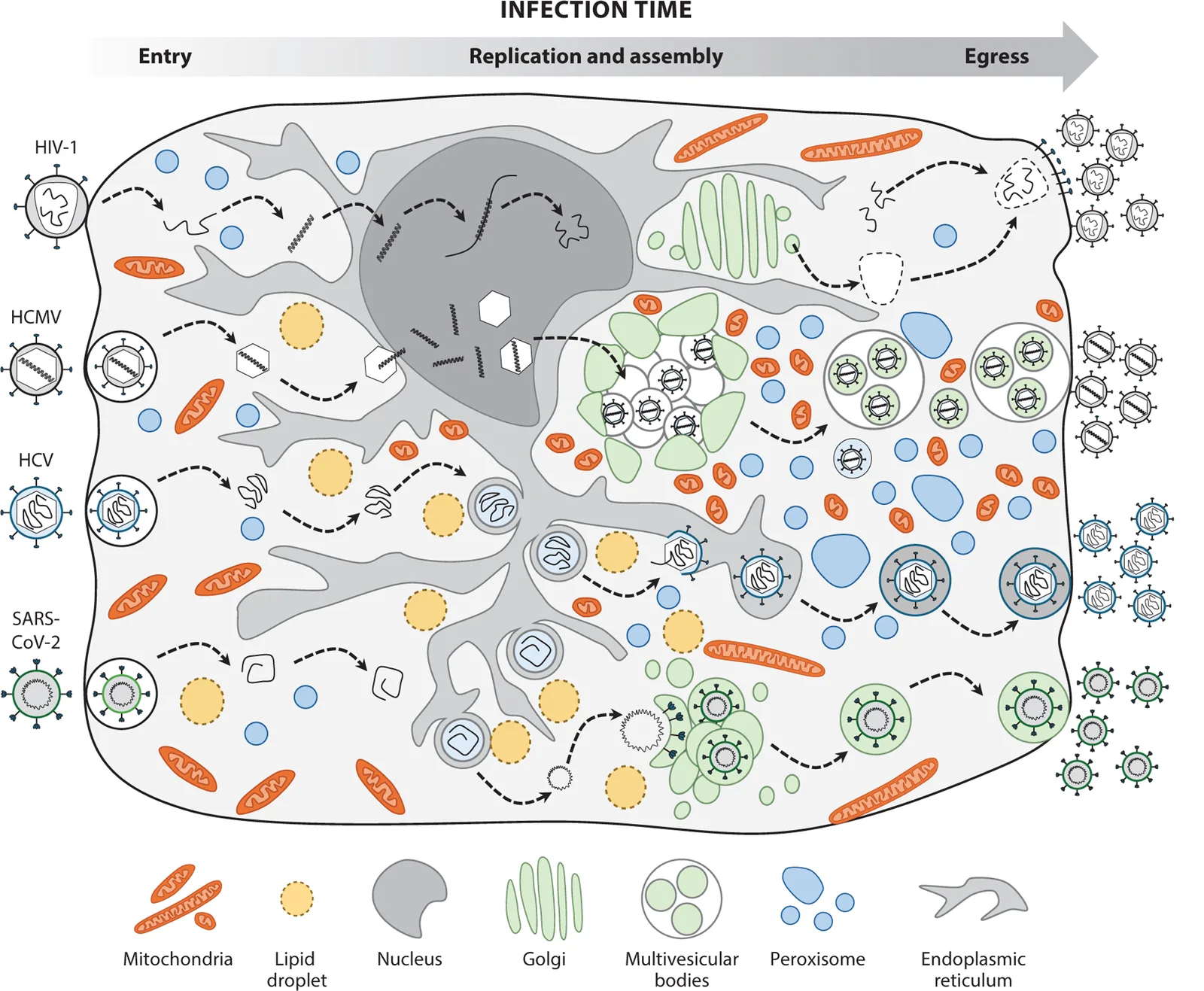
Viruses temporally regulate organelle structure-function throughout their replication cycle. This schematic shows the virus replication cycles of HIV-1 (*top*), HCMV (*second from top*), HCV (*second from bottom*), and SARS-CoV-2 (*bottom*) as well as how these viruses uniquely alter organelle structure and function. Virus replication proceeds from left to right, encompassing viral entry, replication and assembly, and egress. A legend is provided at the bottom of the figure for identifying each individual organelle. Abbreviations: HCMV, human cytomegalovirus; HCV, hepatitis C virus; HIV-1, human immunodeficiency virus type 1; SARS-CoV-2, severe acute respiratory syndrome coronavirus 2.

Given that organelles represent lynchpins of fundamental cellular processes, it is not surprising that organelle dysfunction is associated with a panoply of disease states. This is exemplified by viral infections (Figure 1).As obligate intracellular parasites, most viruses use organelle remodeling mechanisms to control cellular metabolism, immune signaling, and apoptosis. In this review, we discuss how viral infections alter organelle number, morphology, and composition. We further review how these distinct organelle remodeling events are achieved from a molecular standpoint, as well as their known or potential functions in virus replication. Considering the complexity of pro- and antiviral organelle remodeling events, we are framing our discussion from a virus perspective, focusing on the proviral remodeling of mitochondria, peroxisomes, the plasma membrane, lipid droplets, the secretory system, and the endocytic pathway.

## MITOCHONDRIAL REMODELING DURING INFECTIONS

Mitochondria are, perhaps, the most studied organelles with regard to organelle remodeling (12) and, as such, have well-defined structure-function relationships for different shape transitions. Mitochondria are composed of two membranes, with the inner membrane folded into convoluted cristae that house the electron transport chain and promote cellular respiration. During homeostasis, these pliant organelles balance fission, fusion, and degradation through mitophagy to maintain a healthy population (13–15). However, in response to diverse stimuli, including viral infections, the balance between these three processes can be shifted, resulting in altered mitochondria shape and function (16–22). Mitochondria regulate and carry out many essential cellular processes, including cellular respiration, innate immune signaling, apoptosis, lipid oxidation, reactive oxygen species generation, and calcium homeostasis. Given that many of these pathways can be toggled by altering mitochondria fission, fusion, or degradation, mitochondria are remodeled during viral infections (23–27).

### Mitochondrial Fission and Fragmentation

**Figure 2:**
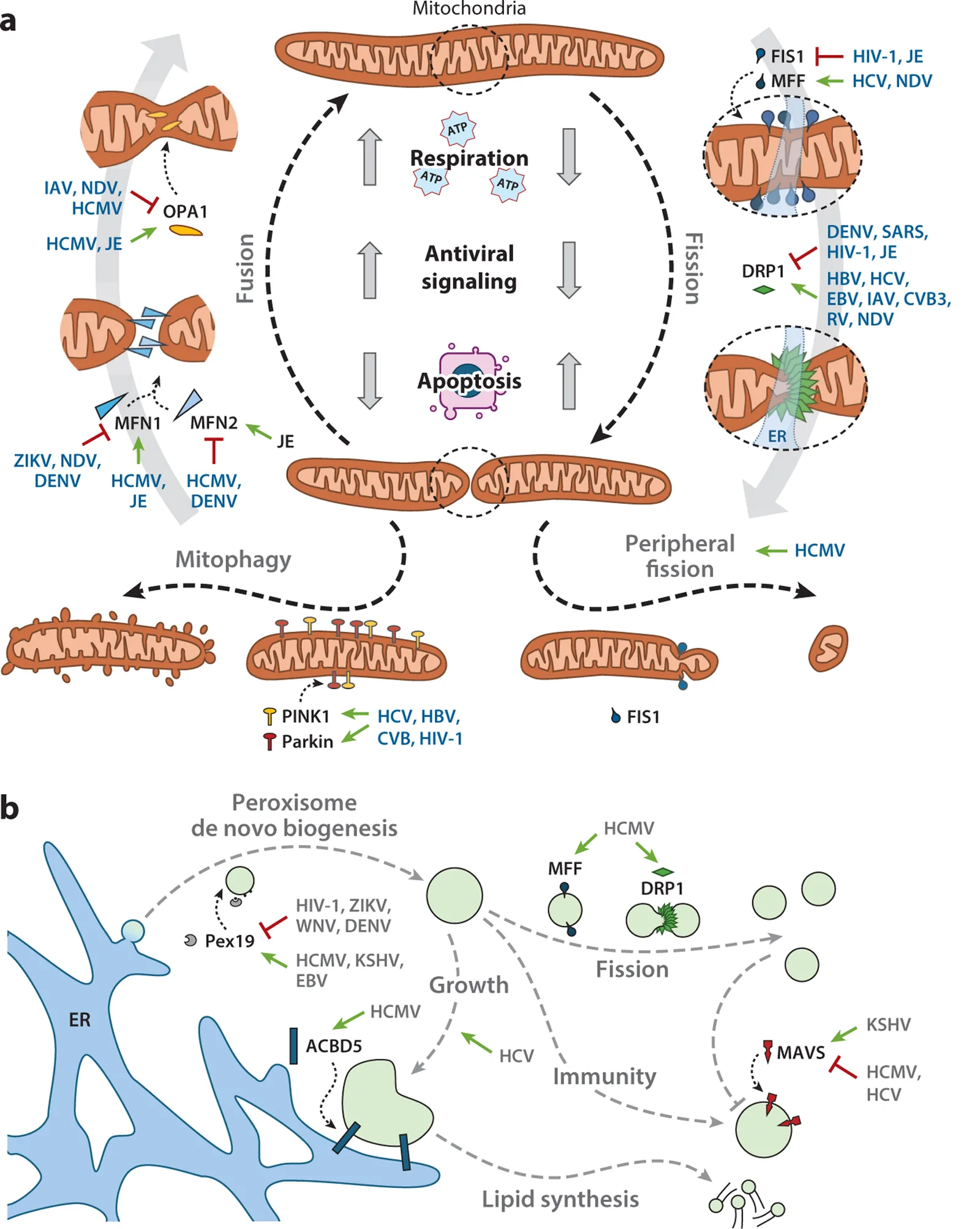
Viruses remodel mitochondria and peroxisomes to toggle metabolism, immune signaling, and cell survival. (*a*) Viruses promote and disrupt mitochondria dynamics by targeting different stages of mitochondria fission, fusion, and degradation through mitophagy. This schematic displays the continual clockwise cycle of mitochondria fission and fusion promoted by host proteins. Red and green arrows indicate viruses found to disrupt or activate the indicated mitochondrial factor, respectively. (*b*) Viruses interfere with different stages of peroxisome biogenesis and growth to either promote or suppress peroxisome-dependent functions. Red and green arrows indicate viruses that disrupt or promote the indicated host protein, respectively. Abbreviations: CVB, Coxsackie virus B; DENV, dengue virus; EBV, Epstein-Barr virus; ER, endoplasmic reticulum; HBV, hepatitis B virus; HCMV, human cytomegalovirus; HCV, hepatitis C virus; HIV-1, human immunodeficiency virus type 1; IAV, influenza A virus; JE, Japanese encephalitis; KSHV, Kaposi’s sarcoma-associated herpesvirus; MAVS, mitochondrial antiviral signaling protein; NDV, Newcastle disease virus; RV, rotavirus; SARS, severe acute respiratory syndrome; WNV, West Nile virus; ZIKV, Zika virus.

Viruses can dysregulate the well-choreographed and temporally tuned processes that underlie mitochondrial fission (Figure 2a). In an uninfected cell, fission begins with ER tubules marking a future site of fission (7,28) that then recruits cytoplasmic fission factors such as MFF and Fis1 (29, 30). These proteins in turn recruit the dynamin GTPase Drp1, which forms an oligomeric ring around the mitochondria (31). Drp1 GTP hydrolysis causes this ring to constrict, thus inducing scission of both mitochondrial membranes. Mitochondrial fission is commonly upregulated during viral infection to promote mitophagy or to impair innate immune signaling and respiration. As a lynchpin for this process, Drp1 is commonly targeted during viral infection to either induce or prevent mitochondrial fission. For example, infections with both hepatitis C virus (HCV) and hepatitis B virus (HBV) activate Drp1 through serine 616 phosphorylation, concurrent with the upregulation of mitophagy (25, 26). Similarly, the influenza A virus (IAV) protein PB1-F2 promotes Drp1-dependent mitochondrial fission and mitophagy, which impairs innate immune signaling (24, 32). This effect seems to be context dependent, however, given that infection with a different strain of IAV in epithelial cells instead promotes mitochondrial elongation (33). Latent infection with Epstein-Barr virus (EBV) also induces Drp1 S616 phosphorylation, which has been linked to poor prognosis for EBV-associated cancer (34).

Not all viruses induce mitochondrial fragmentation through Drp1, however. Human cytomegalovirus (HCMV) induces fragmentation independently of Drp1 (35, 36) by promoting peripheral fission concurrently with the suppression of fusion (37). Mitochondrial progenies derived from peripheral fission were further shown to be protected from mitophagy and result in elevated bioenergetic output through a mechanism leveraging mitochondria-ER encapsulations and inter-mitochondria membrane contacts (37). This alternative pathway for fragmentation may explain why HCMV infection promotes mitochondrial respiration (38–40) while other viral infections that induce fragmentation (e.g., HBV, HCV) instead disrupt respiration.

### Mitochondrial Fusion

Similarly to fission, mitochondrial fusion is a stepwise process that viruses can disrupt or enhance to promote their replication (Figure 2a). To fuse, mitochondria must coordinate both innerand outer-membrane fusion. While linked, these events rely on distinct sets of proteins. Outer-membrane fusion is dependent on inter-mitochondrial homo- and hetero-typic interactions between MFN1 and MFN2 (12). However, inner-membrane fusion is instead controlled by Opa1, which is also a critical regulator of mitochondrial ultrastructure and respiration (41). Mitochondrial fusion is thought to facilitate the exchange of biomolecules between mitochondria that, through complementation, can help to repair damaged mitochondria, thus promoting mitochondrial respiration and cell survival (14). Additionally, mitochondrial fusion has been suggested to promote innate antiviral signaling pathways by inducing the oligomerization of mitochondrial antiviral signaling protein (MAVS) (12, 42). Further supporting Drp1 as a critical regulator of mitochondrial shape, size, and number, both dengue virus (DENV) and severe acute respiratory syndrome (SARS) induce mitochondrial hyperfusion through Drp1 inhibition, as evidenced by decreased Drp1 abundance and S616 phosphorylation, with a concurrent increase in mitochondrial respiration (23, 43). Suppression of mitochondria fission was shown to be important for DENV replication, potentially due to the pro-survival effect of mitochondrial fusion or the increased respiration that could help to fuel virus replication and assembly (23). While mitochondrial fusion is thought to activate innate antiviral signaling pathways by promoting MAVS oligomerization, several viruses that induce mitochondrial fusion (e.g., DENV, SARS) appear to circumvent this by directly inhibiting or degrading MAVS (43, 44).

### Mitophagy

To modulate mitochondrial function and survival, some viruses have acquired mechanisms to dysregulate mitophagy-mediated mitochondrial degradation (Figure 2a). In homeostatic cells, the maintenance of a healthy population of mitochondria involves the mitophagy of aging and dysfunctional mitochondria. Mitophagy serves dual homeostatic roles within a cell by acting as both a quality control mechanism and a method for recycling intracellular components. From a molecular standpoint, mitophagy is triggered when damaged mitochondria lose their membrane potential, resulting in the sequential recruitment of the proteins PINK1 and Parkin (45). Parkin-mediated ubiquitylation of mitochondrial proteins then promotes the formation of an isolation membrane around the damaged mitochondria, which subsequently fuses with a lysosome (45). Mitophagy can also be triggered by cells in response to hyperinflammation as a mechanism for suppressing NLRP3 inflammasome activation. Mitophagy dysregulation is linked to pathogenesis in several disease states, including Parkinson’s disease, several cancers, and viral infection (46, 47). The enterovirus Coxsackievirus B3 (CVB3) induces mitophagy in a range of cell types, which was found to be important for virus-induced suppression of the host interferon response and promotion of viral replication (48). Highlighting the connection between different mitochondrial remodeling processes, CVB3 induces mitophagy by upregulating Drp1-dependent mitochondria fragmentation (48). Induction of mitophagy is also a common method for preventing cell death during an infection, as seen for a range of DNA and RNA viruses (46).

In summary, due to their prevalent roles in metabolism, immune signaling, and cell death, mitochondria are common targets during viral infections. As such, virus-induced mitochondrial dysfunction has also been associated with the progression and development of diseases such as myocarditis and cancer (34,49). Furthermore, Drp1 inhibition is under investigation as a potential strategy for preventing the spread and pathogenesis of select viruses such as CVB3 and Zika virus (ZIKV) (49, 50).

## PEROXISOMES

Although peroxisomes were first described in the 1950s, the full scope of their importance to cellular metabolism and immune signaling did not transpire until the past decade. These organelles were first identified for their ability to convert reactive hydrogen peroxide into water via the enzyme catalase (51). Since then, peroxisomes have also been identified as important factors for the breakdown and biosynthesis of different fatty acid species as well as for immune signaling (52, 53). As such, both pro- and antiviral roles have been identified for peroxisomes in different viral infections (54, 55).

### Size and Number

Given that peroxisomes regulate cellular processes that can be beneficial or detrimental to virus replication, several viruses modulate peroxisome functions by regulating their abundance (Figure 2b). Peroxisomes can proliferate through fission of existing peroxisomes or de novo synthesis from ER- and mitochondria-derived membranes (56, 57). Aside from the peroxisome-specific protein Pex11β, the process of peroxisome fission shares machinery with mitochondria and occurs through a similar mechanism. Conversely, de novo synthesis is orchestrated by a group of peroxisomal proteins, termed peroxins, which mediate membrane budding from the ER or mitochondria. Peroxisomes can also regulate their size, a process linked to the ER-peroxisome MCS protein ACBD5 (9, 58). The processes regulating peroxisome size and number appear to be coordinated such that ACBD5 overexpression increases peroxisome size while reducing their number, whereas ACBD5 knockdown decreases peroxisome size while increasing their number (11).

Decreased peroxisome numbers were observed during several viral infections, possibly to inhibit their role in immune induction (55). Human immunodeficiency virus type 1 (HIV-1) infection induces the formation of several host microRNAs that target four key peroxisome biogenesis proteins, resulting in decreased peroxisome numbers and activity (59).Similarly, decreased peroxisome numbers and suppressed functions were found during infections with several flaviviruses, namely, ZIKV, DENV, and West Nile virus (WNV) (60, 61). Upon infection, viral capsid proteins bind to the peroxisome protein Pex19, which correlates with decreased peroxisome numbers and immune induction (60, 61). Indeed, increased peroxisome proliferation was observed to decrease ZIKV replication, concurrently with upregulated innate immune signaling (60).

### Lipid Metabolism

Peroxisomes can also serve as important factories for lipid synthesis during viral infection. Infections of several herpesviruses—herpes simplex virus type 1 (HSV-1), HCMV, and Kaposi’s sarcoma-associated herpesvirus (KSHV)—cause peroxisome proliferation (62, 63). HCMV and HCV can also induce the formation of a subset of enlarged and irregularly shaped peroxisomes (63, 64). For HCMV, peroxisome biogenesis and the production of peroxisome-derived lipids called plasmalogens were shown to be required for virion secondary envelopment and efficient virus production (63). Separately, plasmalogens were also found to be enriched in the HCMV envelope (65), suggesting that peroxisome morphology and number may be altered to promote the production of these lipids for incorporation into the virion. Peroxisomal proteins involved in lipid metabolism were also found to be important for maintenance of KSHV latency, with loss of these proteins resulting in aberrant cell death (62). Because these alterations to peroxisome dynamics can activate innate immune signaling, HCMV and HCV seem to mitigate these antiviral effects by directing cleavage of the peroxisome resident MAVS (66, 67).

While studies characterizing peroxisome remodeling during viral infections remain limited, peroxisomes can clearly have profound effects during a wide range of viral infections through their functions in immune signaling and lipid metabolism.

## PLASMA MEMBRANE

At the cell surface, the plasma membrane provides a physical barrier from the surrounding environment and controls import and export of molecules. The plasma membrane also houses a dynamic array of proteins and lipids that participate in fundamental cellular signaling events both during homeostasis and in response to stress. Unsurprisingly, viruses interact with this organelle during both entry and egress. Indeed, the plasma membrane is extensively remodeled during many infections to promote viral entry, assembly, and egress, as well as to alter cell signaling pathways.

### Lipid Rafts and Viral Budding

**Figure 3:**
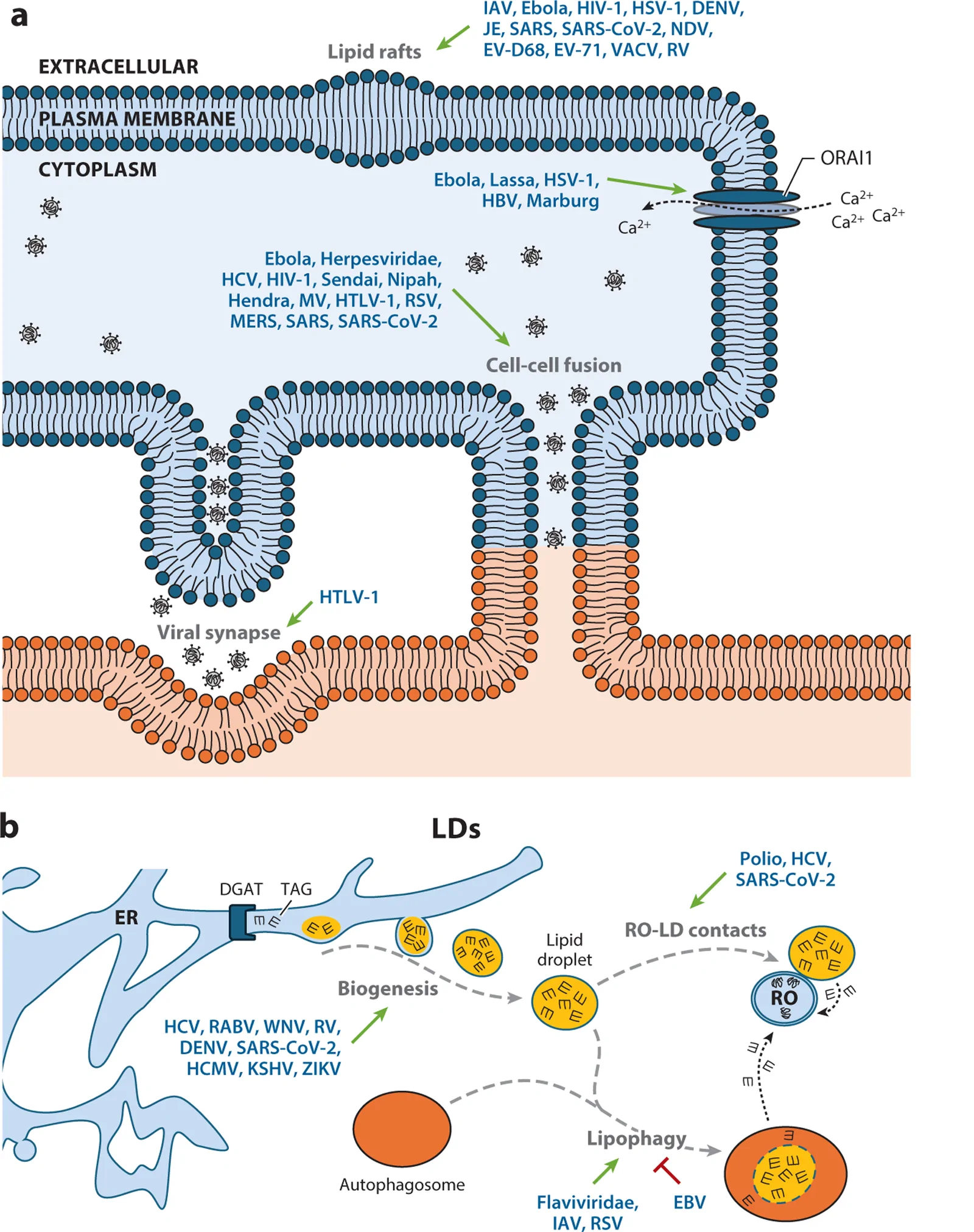
Viruses target the plasma membrane and LDs to promote virion replication, assembly, and spread. (*a*) This schematic indicated how viruses remodel the plasma membrane to promote their replication. Some viruses promote the formation of lipid rafts for assembly and egress (*top*), while others recruit host proteins to the plasma membrane (*right*). To facilitate cell-to-cell spread, some viruses induce cell-cell fusion and/or the formation of viral synapses (*bottom*). Green arrows indicate processes promoted by specific viruses. (*b*) This schematic shows how viruses target LD biogenesis, degradation, and localization to promote replication. Green arrows indicate what processes specific viruses promote, while red lines indicate processes suppressed by specific viruses. Abbreviations: DENV, dengue virus; Epstein-Barr virus; ER, endoplasmic reticulum; EV-71, enterovirus 71; EV-D68, enterovirus D68; HBV, hepatitis B virus; HCMV, human cytomegalovirus; HCV, hepatitis C virus; HIV-1, human immunodeficiency virus type 1; HSV-1, herpes simplex virus type 1; HTLV-1, human T-lymphotropic virus 1; IAV, influenza A virus; JE, Japanese encephalitis; KSHV, Kaposi’s sarcoma-associated herpesvirus; LD, lipid droplet; MERS, Middle East respiratory syndrome; MV, measles virus; NDV, Newcastle disease virus; RABV, rabies virus; RO, replication organelle; RSV, respiratory syncytial virus; RV, rotavirus; SARS, severe acute respiratory syndrome; SARS-CoV-2, severe acute respiratory syndrome coronavirus 2; TAG, triacylglycerol; VACV, vaccinia virus; WNV, West Nile virus; ZIKV, Zika virus.

Stretching back to the 1970s, several viruses have been recognized for their ability to concentrate viral proteins at the host cell plasma membrane. For example, IAV assembles new virions at lipid rafts, which are microdomains within the plasma membrane that are enriched with cholesterol, sphingolipids, and proteins (68–70) (Figure 3a). Expression of the IAV hemagglutinin (HA) promotes the expansion of these domains, forming a platform for efficient virus assembly (70). Lipid rafts can also facilitate the egress of several other viruses, including HIV-1 and Ebola virus (71). These virus-induced alterations to the plasma membrane can change the biophysical properties of the membrane (i.e., charge and fluidity). IAV infection promotes a negative charge on the inner leaflet of the plasma membrane, along with increased lipid packing and a concomitant decrease in membrane protein dynamics, all of which are characteristics of lipid rafts (72). Such alterations in membrane dynamics could help recruit and concentrate viral proteins and promote membrane budding.

### Viral Entry and Cell Signaling

In addition to recruiting viral proteins, several viruses have been shown to concentrate host factors at the plasma membrane. HIV-1 virion binding to a host cell was found to subsequently recruit host coreceptors to the initial binding site (73). Similar strategies have been observed during infections with KSHV, CVB4, and enterovirus D68 (EV-D68) (74).

Viral recruitment of host proteins to the plasma membrane can also dysregulate host signaling pathways. Infection with hemorrhagic fever viruses (e.g., Ebola) was shown to activate the host plasma membrane calcium channel ORAI1, which subsequently increases calcium influx from the extracellular environment (75) (Figure 3a). This increase in cytoplasmic calcium was shown to be important for the formation and production of infectious virions. The PI3K-Akt pathway is also regulated at the plasma membrane, playing a critical role in diverse viral infections (76). Alphaviruses activate PI3K, resulting in the conversion of PIP2 to PIP3 at the plasma membrane, and ultimately facilitating the downstream recruitment and activation of Akt (77). It is not surprising that many viruses target Akt given that it regulates different core cellular pathways, including autophagy, metabolism, translation, and cell proliferation (76). Indeed, suppression of this pathway has been shown to dampen virus replication during both DNA and RNA virus infections, such as vaccinia virus (VACV), alphaviruses, and herpesviruses (76–78).

### Cell-Cell Virus Spread

As an alternative to entering a cell from the extracellular milieu, some viruses also encode mechanisms for promoting cell-cell fusion, thus allowing for the direct transfer of virions from an infected donor cell to an uninfected target cell (79) (Figure 3a). Many enveloped viruses use viral fusogens that promote viral entry for cell-cell fusion, e.g., the spike (S) protein of coronaviruses (80). Not only does cell-cell fusion aid in the dissemination of a virus, but also the resultant multinucleated cells—syncytia—have been correlated with increased virion production, inflammation, and even oncogenesis (81–83). Cell-cell spread does not always require syncytia formation, however. In the case of human T-lymphotropic virus type 1 (HTLV-1), infection causes the formation of virological synapses, where discrete junctions form between donor and target cells, facilitating viral transmission (84) (Figure 3a). Potentially related to the low infectivity of this virus, cell-cell contact is required for infection (85).

Altogether, the plasma membrane plays a crucial role in all virus replication cycles. As such, prevention of virus-induced plasma membrane remodeling, such as through the disruption of lipid rafts, is considered a promising antiviral strategy (49).

## LIPID DROPLETS

While this review has so far focused on membrane-bound organelles, an increasing number of organelles that lack a traditional lipid bilayer membrane are being discovered and identified as important factors for virus infection (86–88). For one, lipid droplets are key regulators of cellular metabolism and homeostasis that are remodeled during many different infections (89, 90). These organelles consist of a hydrophobic core enclosed by a monolayer of phospholipids and proteins. Lipid droplets are dynamic organelles exhibiting context-dependent changes in size, number, distribution, and composition (89,91). Lipid droplet biosynthesis can help alleviate cellular stress and lipid toxicity by sequestering circulating lipids and storing them for later use (89). Conversely, the breakdown of lipid droplets by autophagic machinery frees up the neutral lipids stored within. Beyond their roles in lipid homeostasis, lipid droplets have also been implicated in cellular detoxification, apoptotic regulation, and immune signaling (89–91). Indeed, the antiviral protein viperin localizes to lipid droplets and enhances the interferon response (92).

### Lipid Droplet Biogenesis and Growth

Many viruses promote an expansion of the lipid droplet population to obtain the lipids necessary for their replication and assembly (Figure 3b). Lipid droplets are synthesized de novo from the ER through a multi-step process. Biogenesis begins with the enzymes DGAT and ACAT, which produce triacylglycerols (TAGs) and cholesterol esters, respectively, two lipids that make up the bulk of the lipid droplet core. As these lipids accumulate, they coalesce, first forming a lens-like structure and then ultimately budding from the ER. The resultant lipid droplet consists of acore of TAGs and cholesterol esters surrounded by a phospholipid monolayer, taken from a single leaflet of the ER bilayer membrane. Following formation, lipid droplets become decorated with several different proteins, including the lipid droplet-resident perilipins (PLIN1–5). These proteins play important roles in modulating lipid droplet dynamics and function.

Lipid droplet biogenesis is important for the efficient replication of many viruses, including flaviviruses, coronaviruses, enteroviruses, and rotaviruses (90, 93, 94). For example, rotavirus infection was found to promote formation of lipid droplets, which were further identified as sites for rotavirus assembly (94). Furthermore, inhibition of DGAT or ACAT as well as the dispersal of lipid droplets into numerous microstructures was found to reduce rotavirus titers over 100-fold (94). Similarly, severe acute respiratory syndrome coronavirus 2 (SARS-CoV-2) infection of several different cell types was found to cause lipid droplet accumulation and colocalization with viral proteins (93). DGAT inhibition was further found not only to decrease virus replication but also to dampen the SARS-CoV-2-induced inflammatory response (93). These results suggest that lipid droplets may play dual roles in inflammatory viral infections, both by providing the lipids for assembly and by mediating proinflammatory signaling.

Increased lipid droplet biogenesis not only serves to promote virus replication but also can be an important component of the antiviral host response (95, 96). Decreasing lipid droplet mass was found to stunt interferon production, while promoting lipid droplet formation prior to infection with ZIKV or HSV-1 was found to decrease virus replication (95, 96). These findings suggest that virus-induced lipid droplet dysregulation must be temporally controlled to avoid activation of the antiviral response. Lipid droplet–induced immune signaling is likely stunted in many viral infections by suppressing downstream signaling molecules, such as STING, which is activated by viperin (87, 97).

### Lipid Droplet Contacts

Lipid droplets form MCSs with several different organelles, including the ER, peroxisomes, mitochondria, autophagosomes, and, in the case of viral infection, replication organelles (ROs) (98) (Figure 3b). Since their discovery more than 50 years ago (99), MCSs have been recognized as important regulators of viral replication (100). Similarly to many cellular processes during viral infections, MCSs are restructured by both the host and the virus during an infection and have established roles in viral entry (101), egress (102), and lipid distribution (103). During poliovirus infection, viral proteins first directly recruit lipid droplets to ROs (104). Then, additional viral proteins promote the transfer of fatty acids out of lipid droplets so that the former can be used for phospholipid synthesis and, subsequently, become enriched in the viral envelope (98, 104). Fatty acids from lipid droplets can also be obtained through lipophagy, or the targeted breakdown of lipid droplets. During DENV infection, viral proteins promote MCSs between lipid droplets and autophagosomes, resulting in lipophagy and the subsequent release of lipid precursors that can be used for replication and assembly (105).

In summary, lipid droplets are dynamic organelles that serve both pro- and antiviral roles in virus replication. Lipid droplets represent a major storage location for lipids that can be used to produce the viral envelope Consequently, pharmacological inhibition of host lipid droplet biosynthesis machinery has an antiviral effect on several flaviviruses and may represent a broad-spectrum therapeutic strategy (106).

## THE SECRETORY PATHWAY

The secretory pathway consists of several cooperative organelles that assist in the production, maturation, and localization of proteins (107). Here, we focus on the roles of the ER and the Golgi apparatus during viral infections. The ER consists of a membranous web of interconnected tubules and sheets that stretches across the entire cell. By contrast, the Golgi is composed of several discrete stacks or cisternae that exchange material by using vesicular transport. During their cytoplasmic translation, transmembrane, secretory, and luminal proteins are first targeted to the ER membrane or lumen (108). These proteins are then trafficked to the Golgi, where they can mature and be sorted to their next destination. As obligate intracellular pathogens, all viruses rely on their host cell for the synthesis of host and viral proteins. As such, the secretory pathway is a critical target during viral infection and is commonly reorganized to promote virus replication.

### Replication Organelles

**Figure 4:**
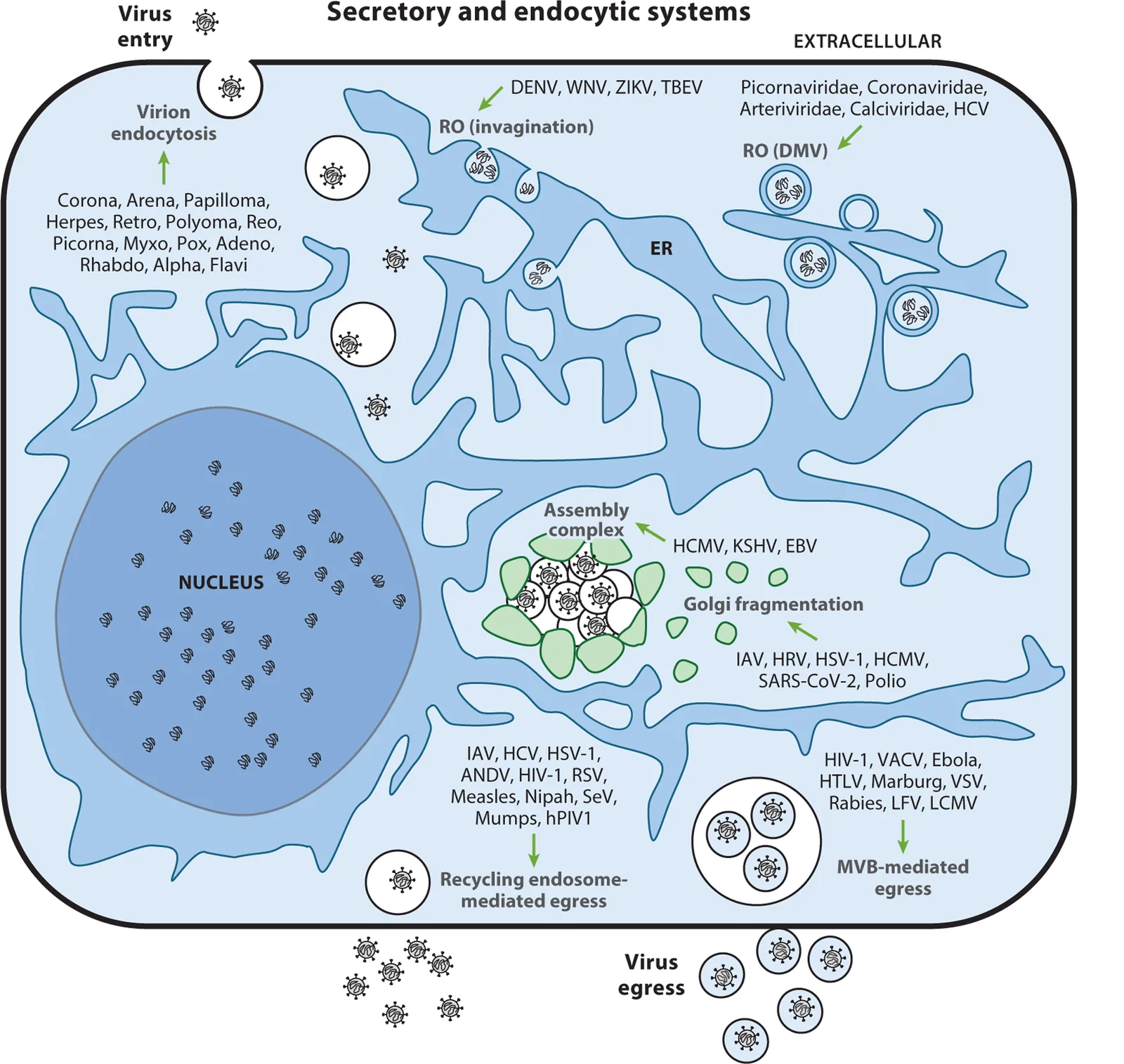
The secretory and endocytic systems are remodeled during virus infections to promote virus entry, replication, and egress. The endocytosis pathway (*top left*) is recruited by a diverse range of virus families to facilitate virus entry. ROs (*top right*), of either the invagination or DMV type, are commonly created during RNA virus infections to create a cytoplasmic pocket in which the virion can replicate and assemble. Several DNA viruses also usurp host membranes to assembly in the cytoplasm, forming an assembly complex (*center*). The Golgi apparatus is targeted during some viral infections to modulate protein production (*center*). To egress from the cell, some viruses remodel and hitchhike on recycling endosomes or MVBs destined to be released into the extracellular space (*bottom*). Abbreviations: ANDV, Andes virus; DENV, dengue virus; DMV, double-membrane vesicle; EBV, Epstein-Barr virus; ER, endoplasmic reticulum; HCMV, human cytomegalovirus; HCV, hepatitis C virus; HIV-1, human immunodeficiency virus type 1; hPIV1, human parainfluenza virus type 1; HRV, human rhinovirus; HSV-1, herpes simplex virus type 1; HTLV, human T-lymphotropic virus 1; IAV, influenza A virus; KSHV, Kaposi’s sarcoma-associated herpesvirus; LCMV, lymphocytic choriomeningitis virus; LFV, Lassa fever virus; MVB, multivesicular body; RO, replication organelle; RSV, respiratory syncytial virus; SARS-CoV-2, severe acute respiratory syndrome coronavirus 2; SeV, Sendai virus; TBEV, tick-borne encephalitis virus; VACV, vaccinia virus; VSV, vesicular stomatitis virus; WNV, West Nile virus; ZIKV, Zika virus.

Most RNA viruses replicate and assemble their genomes within the cytoplasm. To avoid detection by host antiviral defenses during this delicate stage of the virus replication cycle, many RNA viruses reorganize and concentrate cellular proteins and organelles into viral factories or ROs (109) (Figure 4a). These specialized structures often consist of viral proteins surrounded by organelles, which supply metabolites and serve as physical barriers (104, 110). ROs can be formed from a range of organelles, including mitochondria, peroxisomes, the ER, the Golgi, lipid droplets, lysosomes, and endosomes. Perhaps due to the expansive nature of the secretory pathway and the fact that the ER and Golgi are major sites for protein translation and export, they are common sites for cytoplasmic virus replication (110).

ROs can be categorized into two main groups based on their structure—invaginations and double-membrane vesicles (DMVs) (111, 112). Invagination-type ROs are formed during infections with DENV and togaviruses, among others (111, 113). These structures are formed by the negative (inward) ER curvature, which leads to inward budding and the formation of a pocket that extends into the ER lumen (Figure 4a). For DENV, and perhaps other flaviviruses, these structures are commonly found to be adjacent to virion containing ER cisternae, ostensibly to ensure that nascent viral RNAs can be efficiently packaged into new virions (113). By creating a semi-enclosed space with a small opening (∼11 nm), the RNA replication machinery can be concentrated while still allowing space for the exchange of metabolites and the release of mature viral RNAs. Furthermore, hiding RNAs within these structures also helps prevent detection by innate immune defenses.

The second archetypal RO is the DMV, which serves as a site of replication for many positive-sense RNA viruses, including HCV, poliovirus, and coronaviruses (112) (Figure 4a). DMVs have been found to be formed through two main pathways. In one pathway, a spherule first buds from a membrane and then stretches and thins before wrapping back upon itself to form an enclosed DMV. This is a strategy used by picornaviruses (114). Other viral families such as the nidoviruses, including arteriviruses and coronaviruses, instead form a thin, elongated protrusion from the membrane that subsequently curls back on itself to form the DMV while still remaining connected to the parent membrane (112, 115). These DMVs can either stay connected to the parent organelle or undergo fission to become free-floating. While ROs that remain connected to the parent organelle can still obtain materials for continued replication through this umbilical link, fully enclosed DMVs have no evident means of material exchange during viral infections. It is unclear if active replication is occurring in these closed DMVs, potentially aided by molecular pores that cannot be discerned by electron microscopy, or whether these structures instead serve to sequester surplus viral RNAs and thus limit immune activation.

The formation of cytoplasmic assembly complexes is, however, not exclusive to RNA viruses (116). Several herpesviruses, including HCMV, KSHV, and EBV, have also been found to reorganize organelles into a viral assembly complex (vAC) (117–119) (Figure 4a). During HCMV infection, the vAC is formed through the reorganization of secretory and endocytic machinery, forming a ring of Golgi that is filled with early endosomes (117). The vAC also acts as a microtubule-organizing center, with the nucleation of new microtubules occurring predominantly at the Golgi-derived vAC (120). Acetylation of microtubules emanating from the vAC was further shown to be important for the architecture of the vAC as well as for nuclear rotation that can promote cellular and intranuclear polarity (120, 121).

### Golgi Reorganization

A common strategy acquired by different viruses for modulating protein production and intracellular trafficking is through the fragmentation and reorganization of the Golgi during infection (Figure 4a). Fragmentation can promote viral replication by dampening the activation of major histocompatibility complexes as well as the secretion of cytokines. Indeed, during HCV infection, the protein immunity-related GTPase M is activated to promote the fragmentation of the Golgi network (122). Pieces of the fragmented Golgi network subsequently colocalize with replicating HCV, presumably to aid in the synthesis of viral proteins (122). IAV infection was also found to induce Golgi fragmentation and dispersal, a phenotype associated with decreased protein export (123). Fragmentation of the Golgi network by IAV was also shown to trigger inflammasome activation, a hallmark of IAV infection (124). Intriguingly, while the picornavirus human rhinovirus 16 (HRV16) also induces Golgi fragmentation, and the individual expression of HRV16 viral proteins 3A and 3B suppressed protein secretion concurrent with this fragmentation, infection with a full-length HRV16 did not disrupt protein secretion (125). These findings suggest that perhaps other HRV16 proteins or host factors help maintain protein secretion despite the fragmented state of the Golgi.

Golgi fragmentation has also been observed during DNA virus infections, including several herpesviruses (126,127). For example, infection with HSV-1 triggers Golgi fragmentation in a cell type–dependent manner (126). Notably, HSV-1 infection in neuronal cells, a primary site for the HSV-1 latency, induces Golgi fragmentation, a phenotype also observed in several neurodegenerative diseases (128). Recently, it was discovered that HCMV also fragments the Golgi network in a cell type–dependent manner, with extensive fragmentation occurring during infection in epithelial cells but not in fibroblasts (129).

### Endoplasmic Reticulum Stress

Diverse viral infections cause ER stress through rearrangement of the ER and Golgi membranes as well as by increasing the production of viral proteins (130). To prevent the accumulation of unfolded or misfolded proteins within the cytoplasm, which would cause ER stress, cells encode the unfolded protein response (UPR) signaling pathway (131). Through three arms (PERK, IRE1α, and ATF6), this pathway can modulate transcription, translation, protein folding, and apoptosis in response to varying degrees of ER stress. UPR activation is particularly prevalent upon infections with viruses that induce extensive ER remodeling and/or are cytopathic (130). While some aspects of the UPR can be antiviral, many viruses have evolved to toggle this pathway to dampen antiviral effects and promote factors that enhance virus replication. For example, DENV infection initially triggers translational attenuation through the PERK arm of the UPR (132). However, as infection progresses, this effect dissipates and the IRE1α and ATF6 arms of the UPR are instead activated, resulting in the increased production of chaperones, which can assist in viral protein folding, and pro-survival factors to prevent apoptosis (132). Some viruses also encode mechanisms to bypass the potentially antiviral effects of the UPR. For example, while the UPR can induce global translation attenuation, the HCV viral genome encodes an internal ribosome entry site (IRES) that is not attenuated, thereby promoting the translation of viral proteins while suppressing that of host proteins through other mechanisms (133). Although initial signaling events activated by the UPR attempt to resolve the ER stress, continued activation of the UPR can lead to apoptosis through IRE1. Indeed, of the 36 different viruses that have been found so far to cause ER stress, 23 were also found to induce or accelerate apoptosis (130). The resultant cell death caused by these infections can be critical in viral spread and pathogenesis.

### Autophagosomes

Born from the ER membranes, autophagosomes are double-membraned structures that represent an intermediate stage of the autophagy degradation system. Autophagosomes are formed by ER tubules elongating and engulfing damaged organelles and materials destined for degradation. Once this structure is sealed, it can fuse with the lysosome, resulting in acidification of the compartment and subsequent recycling of the enclosed materials. Autophagy can be antiviral by directly degrading the viral genome and viral proteins or by stimulating the immune system (134). However, many viruses have evolved to not only evade autophagic clearance but also leverage the autophagic machinery to promote virus replication. For example, the autophagy machinery can be repurposed to aid in the formation of ER-derived DMVs, as autophagosomes are a type of DMV. Thus autophagosomes, or abortive autophagosomes, represent sites of replication for several viruses, including poliovirus and flaviviruses (135, 136). To block the ultimate acidification of these autophagosomes, HCV inhibits lysosome-autophagosome fusion (135).

## THE ENDOCYTIC PATHWAY

The endocytic pathway consists of several different organelles that mediate the uptake, recycling, degradation, and export of materials (137). Materials such as proteins and lipids can be endocytosed at the plasma membrane, where they first enter early endosomes. Early endosomes can then mature into recycling endosomes, which return cargo to the plasma membrane, or late endosomes and ultimately lysosomes, where cargo is degraded. In addition to having different functions, endosome subtypes (i.e., early, late) are generally defined by their morphology and proteome, particularly by which Rab proteins are present or absent. Given that this pathway represents a homeostatic pipeline for entering and leaving cells, viruses have acquired mechanisms to use the endocytic system to facilitate viral entry and egress (101).

### Viral Entry Through Endocytosis

Endocytosis represents a predominant method of entry for many viruses for several reasons (138) (Figure 4a). First, this pathway allows for direct transport from the plasma membrane to a perinuclear space within the infected cell, thus facilitating the transition from entry to viral replication. Second, within endosomes, viruses are better shielded from detection by the host intrinsic immune system (101). Finally, the gradient in ion concentrations and pH as endosomes mature and are trafficked through the cell can serve as an indicator to the internalized virion of its location within the cell. Many viruses leverage the acidification of endosomes for entry and uncoating by encoding pH-dependent membrane fusogens, which cause fusion of the viral envelope with the endosome membrane at low pH, allowing for precise spatiotemporal control of these processes (101, 138, 139). A well-established example is the HA encoded by IAV (140). Virions are also vulnerable at this stage of infection in that a virus cannot replicate unless it escapes from endosomes. For example, Ebola virus, which enters the cell through endocytosis, relies on two-pore channels (TPCs) to induce endosome maturation and acidification (141, 142). Consequently, loss of TPCs causes virions to be trapped in endosomes, thus preventing infection (142).

### Endosomes as Sites of Virus Assembly

Some mammalian and plant viruses construct their ROs from endosomal and lysosomal membranes (Figure 4a). For example, positive-sense RNA viruses from the *Alphavirus* genus modify endosomal and lysosomal membranes, forming cytoplasmic vacuoles where viral genome replication takes place (143, 144). Endosomes have also been found to be important for genome replication at the RO of mouse hepatitis virus, a coronavirus (145). Additionally, the vAC formed during HCMV infection is also composed of early endosomes along with the Golgi (127).

### Trafficking to the Plasma Membrane

Just as branches of the endocytic pathway that import materials into the cell can be co-opted for virus entry, other arms of the endocytic pathway that traffic material to the plasma membrane can be employed for viral assembly and egress (102,137) (Figure 4a).For example, IAV, which assembles at the plasma membrane, relies on the recycling endosome regulatory protein Rab11 for the cytoplasmic transport of viral RNA genome segments (146). Following assembly and maturation in the secretory system, HCV has also been shown to hitchhike on Rab11 recycling endosomes for trafficking to the plasma membrane and subsequent virion release (147).

Multivesicular bodies (MVBs) represent a second pathway within the endocytic system that facilitates viral transit to the plasma membrane (148) (Figure 4a). As the name implies, MVBs consist of an enlarged vesicle filled with smaller vesicles. These structures arise from endosomes and function to both degrade ubiquitinated proteins and promote the release of vesicles through exocytosis. To interact with MVBs, a range of RNA viruses, including retroviruses, rhabdoviruses, filoviruses, arenaviruses, and paramyxoviruses, encode a structural protein that contains a late domain motif (L-domain) (148). The L-domain promotes the assembly, budding, and/or egress of viral particles by facilitating interactions with the host MVB biogenesis machinery, such as ESCRT components (148). In the case of HIV-1, the viral structural protein Gag contains an L-domain that recruits the ESCRT machinery to promote MVB biogenesis and, ultimately, virion budding (149). Usurping MVBs for virion egress is not unique to RNA viruses, however. MVBs are important for virion egress and release during VACV infection. Modified MVBs, termed multiviral bodies, were shown to promote bulk virion release during HCMV infection (150, 151).

In sum, the endocytic pathways are altered and employed by RNA and DNA viruses alike to promote viral entry, egress, and spread. While viruses can benefit from using the endocytic system as a means of travel throughout the cell, on the flip side, they are also vulnerable to entrapment at these stages, as shown for Ebola virus and Middle East respiratory syndrome (MERS) (142, 152).

## CONCLUDING REMARKS AND PERSPECTIVES

In this review, we discussed the predominant mechanisms that viruses use to remodel organelles. While the phrase organelle remodeling may evoke dramatic alterations in organelle shape, we also showed how altered organelle composition, proliferation, and MCS control organelle function during infection. Indeed, all facets of organelle remodeling often converge on the functional tuning of an organelle. We further showed that many of these organelle remodeling events are shared by diverse viruses often separated by millions of years of evolution. For example, mitochondrial fission is induced during infection with both HCMV, a double-stranded DNA virus with a 235-kb genome that replicates over five days, and IAV, a segmented negative-sense RNA virus with a 13.5-kb genome that replicates in less than a day. Reflecting the unique needs of each virus for replication, we also highlighted how some viruses had acquired opposing requirements for organelle functions. For example, while HCV and HCMV infections promote peroxisome enlargement, ZIKV and other flavivirus infections decrease peroxisome abundance.

From these studies, it is evident that the investigation of virus-induced organelle remodeling has helped elucidate viral replication strategies, identify novel therapeutic targets, and uncover basic biology. However, much of the research on virus-induced organelle remodeling has been limited to a relatively small number of viruses that do not represent the extraordinary viral diversity. Even for well-studied viruses, not all organelles have been rigorously analyzed, and these studies frequently focused on a few cell types. Furthermore, it is often difficult to determine the relationship between the remodeling of organelle shape and a change in function during an infection, as well as whether this event is a host response or a virus-modulated process. Thus, organelle remodeling still represents an exciting frontier for uncovering viral replication tactics and pathogenesis. We hope that our review will encourage future studies in this critical and expanding area of research.

## Abbreviations

HCV: hepatitis C virus
HBV: hepatitis B virus
IAV: influenza A virus
HCMV: human cytomegalovirus
DENV: dengue virus
SARS: severe acute respiratory syndrome
CVB3: Coxsackievirus B3
ZIKV: Zika virus
HIV-1: human immunodeficiency virus type 1
WNV: West Nile virus
HSV-1: herpes simplex virus type 1
KSHV: Kaposi’s sarcoma-associated herpesvirus
EBV: Epstein-Barr virus
EV-D68: enterovirus D68
VACV: vaccinia virus
HTLV-1: human T-lymphotropic virus type 1
SARS-CoV-2: severe acute respiratory syndrome coronavirus 2
HRV16: human rhinovirus 16
MERS: Middle East respiratory syndrome
